# Efficacy of a Soft Robotic Exoskeleton to Improve Lower Limb Motor Function in Children with Spastic Cerebral Palsy: A Single-Blinded Randomized Controlled Trial

**DOI:** 10.3390/brainsci14050425

**Published:** 2024-04-25

**Authors:** Zhichong Hui, Weihang Qi, Yi Zhang, Mingmei Wang, Jiamei Zhang, Dong Li, Dengna Zhu

**Affiliations:** 1 Department of Rehabilitation Medicine, The Third Affiliated Hospital of Zhengzhou University, Zhengzhou 450052, China; huizhichong@gs.zzu.edu.cn (Z.H.); qiweihang@gs.zzu.edu.cn (W.Q.); zy1978921771@gs.zzu.edu.cn (Y.Z.); 15136413122@163.com (M.W.); xiaomei6ok@gs.zzu.edu.cn (J.Z.); dongbaoh521@163.com (D.L.); 2 Zhengzhou Key Laboratory of the Prevention and Cure of Cerebral Palsy Children, Zhengzhou 450052, China

**Keywords:** cerebral palsy, soft robotic, robot-assisted therapy, gross motor function, energy expenditure

## Abstract

Purpose: Soft robotic exoskeletons (SREs) are portable, lightweight assistive technology with therapeutic potential for improving lower limb motor function in children with cerebral palsy. To understand the effects of long-term SRE-assisted walking training on children with spastic cerebral palsy (SCP), we designed a study aiming to elucidate the effects of SRE-assisted walking training on lower limb motor function in this population. Methods: In this randomized, single-blinded (outcome assessor) controlled trial, forty children diagnosed with SCP were randomized into the routine rehabilitation (RR) group (N = 20) and the SRE group (N = 20) for comparison. The RR group received routine rehabilitation training, and the SRE group received routine rehabilitation training combined with SRE-assisted overground walking training. Assessments (without SRE) were conducted pre- and post-intervention (8 weeks after the intervention). The primary outcome measures included the 10 m walk test (10MWT) and the 6 min walk test (6MWT). Secondary outcome measures comprised the gross motor function measure-88, pediatric balance scale modified Ashworth scale, and physiological cost index. Results: Both groups showed significant improvements (*p* < 0.01) across all outcome measures after the 8-week intervention. Between-group comparisons using ANCOVA revealed that the SRE group demonstrated greater improvement in walking speed from the 10MWT (+6.78 m/min, 95% CI [5.74–7.83]; *p* < 0.001) and walking distance during the 6MWT (+34.42 m, 95% CI [28.84–39.99]; *p* < 0.001). The SRE group showed greater improvement in all secondary outcome measures (*p* < 0.001). Conclusions: The study findings suggested that the integration of SRE-assisted overground walking training with routine rehabilitation more effectively enhances lower limb motor function in children with SCP compared to routine rehabilitation alone.

## 1. Introduction

Cerebral palsy (CP) is a syndrome of non-progressive brain injury in fetuses or infants from gestation to the neonatal period due to a variety of causative factors. CP is mainly characterized by motor dysfunction and postural abnormalities, accompanied by varying degrees of perceptual, cognitive, and communication disorders and epilepsy [1]. This condition persists throughout the individual’s life and affects their independence and participation in daily and social activities.

CP can present in a variety of ways, with spastic cerebral palsy (SCP) accounting for approximately 70% to 80% of cases [2]. Children with SCP often experience posture and movement abnormalities due to increased muscle tone and persistent primitive reflexes, leading to limitations in walking speed, balance, and specific movements [3,4]. If left unaddressed, these abnormalities can lead to secondary complications, such as muscle contractures and restricted joint range of motion [5]. The ankle joint is particularly susceptible to these complications due to its crucial role in gait mechanics and weight-bearing. Therefore, targeted rehabilitation training focusing on the ankle joint is essential to preserve soft tissue extensibility, maintain joint mobility, and improve overall gait efficiency [6]. Furthermore, children with ambulatory CP often expend more energy during walking than their typically developing peers due to inefficient movement patterns. This increased energy demand can lead to fatigue diminished interest in physical activity [7], and contributes to muscle atrophy.

In recent years, robot-assisted gait training (RAGT) has been recognized as an effective method to improve lower limb motor function in children with SCP [8,9]. Exoskeleton rehabilitation robots commonly used in the market are usually bulky, labor-intensive to wear, and expensive [10]. With the development of soft exoskeleton technology [11], soft robotic exoskeletons (SREs) have emerged as lightweight wearable walking assistive devices. They are more affordable than traditional exoskeleton robots and have the potential to broaden their use in community and home environments. This is crucial for improving the community participation of children with SCP, especially in developing countries. In addition, the SRE assists the wearer’s natural ankle motion by providing auxiliary support to the dorsiflexors and plantarflexors [12,13]. This represents a patient-initiated training mode, where the walking process is completely controlled by the wearer, which may be more motivating for the wearer, especially for children. This assistance contrasts with most traditional exoskeleton robots, which typically dictate the wearer’s movement based on pre-programmed trajectories [14]. Unlike treadmill-based therapies, SREs are mobile and offer users a realistic and unrestricted walking experience, which is crucial for promoting functional recovery and reintegration into daily activities [15]. Studies involving healthy individuals have shown that SREs can effectively apply controlled forces, thereby enhancing the walking economy [16]. In the context of CP, a study has demonstrated significant improvement in the six-minute walk test performance among children using SREs [17]. Research involving stroke patients has also shown promising results. SREs can promptly modify gait functions, reduce the energy expenditure required for walking, and even sustain therapeutic benefits in unassisted walking after the device is removed [12,18,19]. Notably, a multicenter clinical study involving 36 stroke patients who underwent a 5-day SRE-assisted walking therapy reported improvements in their unassisted walking speed [20].

A previous study has demonstrated that SRE could improve motor function in adult subacute stroke patients [21]. To the best of our knowledge, there are no randomized controlled trials evaluating the effect of SRE combined with traditional rehabilitation on lower limb motor function in children with SCP.

The purpose of this study was to evaluate the effects of SRE on lower limb motor function in children with SCP. It was hypothesized that the combination of SRE-assisted training with routine rehabilitation training would be more effective in promoting the improvement of lower limb motor function in children with SCP than routine rehabilitation training alone.

## 2. Materials and Methods

### 2.1. Subjects and Inclusion Criteria

Eligible patients were children with lower limb motor deficits secondary to SCP. These children were required to have the capacity to understand the basic experimental content and whether they had received previous rehabilitation treatment. These patients were recruited from the inpatient rehabilitation center of the Third Affiliated Hospital of Zhengzhou University.

Inclusion criteria: ① Diagnosis of SCP and categorized within levels I to III of the Gross Motor Function Classification System (GMFCS), and the calf triceps muscle spasticity rated as levels I to III on the Modified Ashworth Scale (MAS); ② Aged between 3–10 years, with the ability to understanding instructions and cooperate with the training program; ③ Capability to walk for 6 min, with or without the aid of assistive devices; ④ Absence of orthopedic surgery or botulinum toxin injections within the previous 6 months; ⑤ Guardians’ informed consent obtained.

Exclusion criteria: ① Current use of anti-spasticity medication; ② Presence of significant complications affecting the heart, liver, kidneys, etc.; ③ Severe epilepsy, genetic metabolic diseases, or major skeletal system disorders; ④ Severe coagulation disorders or thrombotic diseases; ⑤ Untreated severe skin lesions on the lower limbs; ⑥ Acute or progressive neurological diseases.

### 2.2. Study Design

This single-center, single-blinded(outcome evaluator), randomized controlled trial was conducted at the Third Affiliated Hospital of Zhengzhou University (China) from January 2023 to June 2023. The trial was approved by the Ethics Committee of the Third Affiliated Hospital of Zhengzhou University (Approval No.: 2022-403-01) and registered in the Chinese Clinical Trials Registry (Identifier: ChiCTR2300067812). All subjects and/or their parents/legal representatives gave written informed consent in accordance with the Declaration of Helsinki.

**Figure 1:**
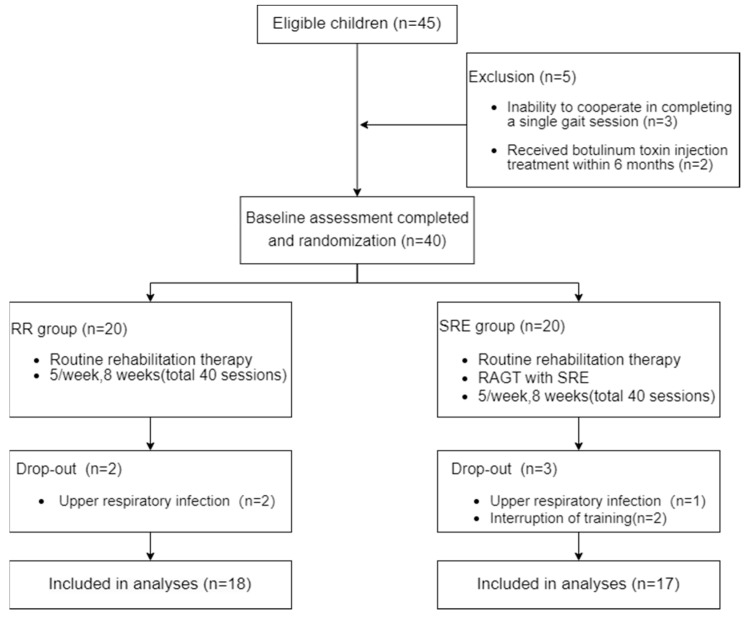
Flow diagram of the study.

All recruited participants were receiving treatment at an inpatient rehabilitation center. The RR group underwent routine rehabilitation training, while the SRE group engaged in both routine rehabilitation training and SRE-assisted overground walking training. The prescription of the total training time of both groups is kept identical. We referenced research designs from previous studies, and positive results are usually observed when the number of training sessions is between 10–40, and the duration of the intervention is between 6–10 weeks [22,23,24]. Therefore, in our study, the intervention was scheduled five times per week over a total of 8 weeks, amounting to 40 sessions. The study protocol timeline is shown in Figure 1.

### 2.3. Sample Size and Randomization

The sample size was determined based on a previous randomized controlled trial investigating robot-assisted walking training in children and adolescents with cerebral palsy, which used a sample size of 24 [25]. To account for a potential 20% dropout rate and achieve a power of 80%, this study recruited 40 participants, with 20 allocated to each group.

Following the initial baseline assessment, subjects were randomly assigned to the RR group or the SRE group in a 1:1 ratio. Randomization was performed using a computer-generated table created by an independent statistician. To ensure allocation concealment, sequentially numbered, opaque envelopes containing the group assignments were prepared by a research assistant not involved in the recruitment or assessment of participants. At the time of registration, eligible new subjects were randomly given an envelope that determined their group.

### 2.4. Soft Robotic Exoskeleton

In this study, Relink-ANK (Yrobot Technology Co., Ltd., Suzhou, China), or “Relink” for short, was used. This is a commercially available SRE based on soft exosuit technology [11]. This particular SRE was chosen for its lightweight design, ease of use, and ability to provide customized assistance to the ankle joint. It consists of an actuator unit and two leg braces supplemented by associated fabrics for improved comfort and fit. The actuator unit houses two electric motors, a central processing unit (CPU), a battery, and a pulley system, and it is fastened to the user’s waist via straps. Leg brackets, designed to attach to the calf and foot, are interconnected with the actuator unit through communication and Bowden cables. These cables facilitate data transfer and motor assistance by transmitting sensor data to the CPU for real-time processing and conveying motor-generated forces to aid ankle motion.

**Figure 2:**
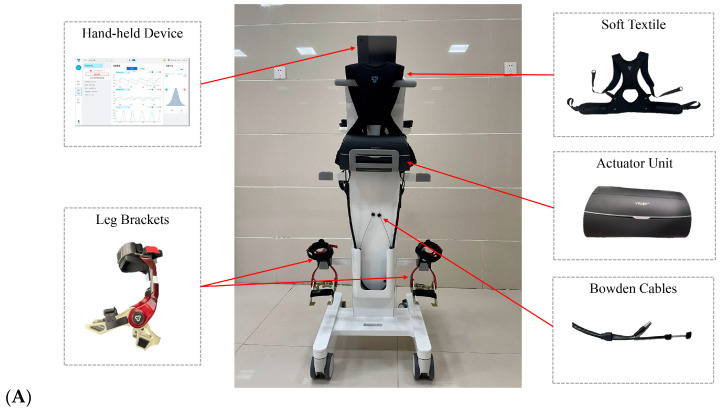

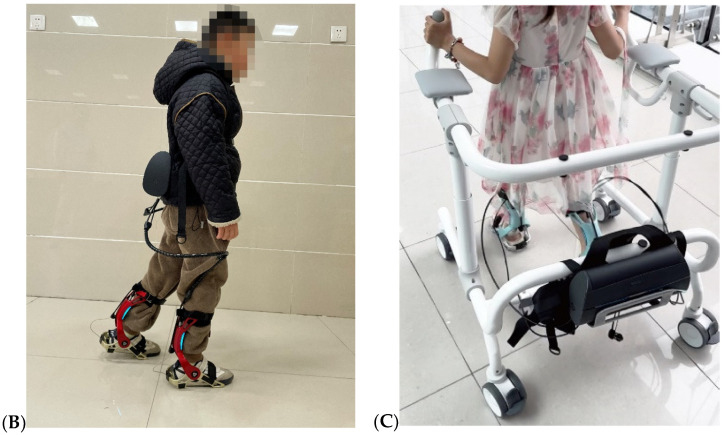
(**A**) Relink-ANK soft robotic exoskeleton, (**B**) SRE-assisted overground walking training, (**C**) SRE-assisted overground walking training with a walking aid.

The entire system weighs approximately 3 kg, with the actuator unit weighing about 2 kg and each leg bracket approximately 0.47 kg. For children who require additional vertical support during walking or cannot bear the weight of the actuator unit, a specially designed walking aid by Yrobot is available. This walking aid is equipped with a foldable chair and height-adjustable handrails to accommodate varying child heights and to ensure a seamless donning/doffing process. See Figure 2 for a visual depiction.

During operation, the Relink utilizes inertial measurement units (IMUs) mounted on the shank and foot brackets to gather kinematic data on the user’s leg and foot movements. These data include parameters such as displacement, orientation, ankle angle, and spatiotemporal aspects of gait. Using this information, Relink dynamically identifies the user’s gait pattern in real-time. This allows for precise adjustments to the tension in the Bowden cables, providing optimal plantarflexion support.

The assistance force is meticulously calibrated to mimic a near-sinusoidal waveform. It initiates just after mid-stance and peaks at or slightly after heel-off, facilitating support during the terminal stance and pre-swing phases of gait. As the user transitions into the initial swing phase, detected by changes in ankle angle and angular velocity, the device gradually reduces plantarflexion support. At this point, dorsiflexion assistance is provided by integrated torsion springs within the leg brackets, enhancing the natural gait motion. The Relink’s adaptability ensures personalized gait assistance for each user, matching their walking patterns for a more natural experience. Designed for ease of use, the Relink can be donned or removed quickly, typically in under a minute, with minimal training for medical personnel or family members. Further enhancing its functionality, the Relink connects to the Yrobot ANK app (Version number: 1.0) via Bluetooth, enabling therapists to remotely access gait analytics and tailor the assistance level as needed.

### 2.5. Intervention

Routine rehabilitation includes physical therapy and other complementary therapies. Physical therapy is the primary therapy and includes proprioceptive neuromuscular facilitation to promote coordinated movement of the child’s extremities, balance training, trunk and joint loading exercises, and postural and motor control training in conjunction with task-oriented training (TOT). Physical therapy was performed five times a week for 8 weeks (two sessions totaling 60 min each).

In addition to this, three treatments were used to provide adjunctive therapy to enhance the effectiveness of physiotherapy. Neuromuscular electrical stimulation (NMES): two electrode pads were placed at the ends of the muscle belly of the spastic muscle for a period of 5 min with a 10 min interval, with a frequency of 1 hertz and a pulse width of 100 milliseconds. Wax therapy: paraffin wax heated to 52 °C–55 °C was wrapped around the skin surface of the patient’s spastic triceps calf muscle. In addition, massage therapy is one of the rehabilitation therapies in Chinese medicine, which uses combined massage techniques such as manipulative massage on spastic muscles to improve spasticity. These three therapies were performed five times a week for 8 weeks (each for 30 min).

For children in the SRE group, one session of physical therapy (a total of two sessions per day of 30 min each) was replaced by one 30 min session of SRE-assisted training, which was done to ensure that the duration of treatment was consistent for both groups. In preparation for the intervention, therapists utilized a tablet application to input each child’s data and meticulously adjust the device’s settings according to individual height and limb length measurements. To address safety concerns and ensure comfort with the technology, a 30 min acclimatization session was held prior to starting the formal training, allowing children to become acquainted with the equipment. During the intervention, participants were encouraged to walk at their own pace along a 30 m pathway for a total of 30 min per session, benefitting from the support of the Relink system. The assistance provided by Relink was gradually reduced if the child’s active motor function improved, thereby tailoring the level of support to the child’s evolving needs. The intensity of the training was designed to challenge the children appropriately, requiring them to exert effort to complete the exercises. Safety remained paramount throughout the intervention, with therapists vigilantly monitoring the participants to promptly identify and address any potential adverse events. This careful oversight ensured that each 30 min session, conducted five times weekly, was executed with the utmost consideration for the children’s safety and comfort, fostering an environment conducive to effective rehabilitation.

### 2.6. Assessment and Outcome Measures

Assessments were conducted by a rehabilitation assessor with over five years of experience, who performed baseline (pre-intervention) and after 8 weeks (post-intervention) evaluations on all children and was unaware of the group assignments. All children were assessed without SRE equipment. The primary outcome measures of this study were the 10 m walk test (10MWT) walking speed and the 6 min walk test (6MWT) walking distance. Secondary outcome measures included clinical assessment scales: gross motor function measure (GMFM), pediatric balance scale (PBS), modified Ashworth scale (MAS) scores, and indicators of energy expenditure during activity: physiological cost index (PCI) values.

#### 2.6.1. 10-Meter Walk Test [26]

The 10MWT is a commonly used clinical assessment of walking ability. Children are instructed to walk at their self-selected walking speed on a 14 m walkway, with markings at the 2 m and 12 m points. The time taken to complete the middle 10 m is measured using a stopwatch and recorded. The test is conducted three times, with a 5 min interval between each trial, and the average speed is calculated.

#### 2.6.2. 6-Minute Walk Test

The 6MWT is a commonly used method to measure walking endurance in children with cerebral palsy [27]. The child walks back and forth along a 30 m walkway for 6 min at their self-selected pace, and the total distance walked is measured. Assistive devices (walkers, orthoses, shoes, etc.) may be used, although none of the children in this study required them for the 6MWT.

#### 2.6.3. Gross Motor Function Measure [28]

The GMFM-88 is the widely used assessment tool for measuring gross motor function in children with cerebral palsy. It consists of five functional domains (A to E) with a total of 88 items. Each item is scored on a four-point scale (0 to 3). This study focused on the raw scores from domains D and E, which specifically assess standing and walking abilities. These domains were chosen as they were most relevant to the intervention being investigated.

#### 2.6.4. Pediatric Balance Scale

The PBS, adapted from the Berg balance scale, is used to assess balance abilities in children with mild to moderate motor impairments. It includes 14 items, each scored from 0 (lowest function) to 4 (highest function), with a maximum score of 56.

#### 2.6.5. Modified Ashworth Scale

The MAS is a simple and easy-to-administer scale that does not require any instruments consisting of six grades: (0, 1, 1+, 2, 3, and 4), which are sequentially assigned scores from 0 to 5. The MAS is used to assess spasticity by measuring the resistance to passive movement in a muscle. Higher scores indicate more severe spasticity.

#### 2.6.6. Physiological Cost Index [29]

The PCI is a common index used to determine the energy consumption of walking [30]. It is calculated as the difference between walking heart rate and resting heart rate, divided by the walking speed. To measure the PCI, children first rested quietly in their seats for 5 min. Their resting heart rate (resting HR) was then measured using a POLAR H10 heart rate chest strap. Next, the children walked back and forth on a 30 m round-trip trail at their self-selected walking speed for 6 min. Walking heart rate (walking HR) was measured after 6 min of walking, and the total walking distance was also recorded. The test was conducted indoors with a room temperature of 20 °C~25 °C. The PCI was then calculated as the difference between walking heart rate and resting heart rate, divided by the walking speed: A higher PCI values indicate higher energy expenditure per unit of walking time. The following formula was used to calculate PCI:

$$PCI(beats/m)=\frac{walking\;HR-resting\;HR}{V(m/min)}$$

### 2.7. Statistical Analysis and Minimal Clinically Important Difference

Statistical analyses were conducted using SPSS 24.0 (IBM Corporation, Armonk, NY, USA) for statistical tests and GraphPad Prism 9.5.0 for graphical representations. SPSS was used for the main statistical analyses due to its comprehensive range of statistical tests, while GraphPad Prism was used to create high-quality figures. Continuous variables were presented as mean [standard deviation, (SD)] while categorical variables were summarized as counts (percentages). No data transformations were performed before analysis.

The Shapiro–Wilk test assessed the normality of the data distribution. Analysis of covariance (ANCOVA) was employed to compare the improvement (post-intervention compared with pre-intervention) in all outcome indicators between the groups, with baseline values used as covariates. Furthermore, paired *t*-test or Mann–Whitney U-test were used to compare varies between pre-and post-intervention. To further explore the possible causes of SRE-induced changes in energy expenditure, we used Pearson and Spearman linear correlation analyses to analyze changes in gross motor function, balance function (GMFM-D, E-zone, and PBS) indices, and changes in energy expenditure indices (PCI). Significance was set at α = 0.05 in all statistical analyses.

The minimal clinically important difference (MCID) is a crucial gauge of clinical relevance. It indicates the smallest change in an outcome measure that is considered meaningful to patients and clinicians. In this study, the clinical significance of observed changes was evaluated against predetermined MCID values. Due to the lack of MICD for 10MWT in children with CP, the MCID value of 0.14 m/s for 10MWT walking speed in adult stroke patients was used as the reference [31]. In 6MWT, the MCID values in patients with CP were determined to be 46 m [32]. In children with grade I–III CP, the MCIDs at large (0.8) effect sizes in GMFM-D and E zones were determined to be 1.8 and 2.6, respectively [33].

## 3. Results

### 3.1. Participants

Thirty-five children completed the study, 18 in the RR group and 17 in the SRE group. Five children withdrew from the study due to personal reasons unrelated to the interventions. Comparative analysis of demographic characteristics (Table 1) and baseline assessment metrics prior to treatment (Table 2) revealed no statistically significant differences (*p* > 0.05) between the groups. During the SRE training, there were no reports of safety issues or side effects in any of the children.

### 3.2. Primary Outcome Measures

Following an 8-week treatment, both the RR and SRE groups exhibited significant enhancements in their 10MWT walking speed and 6MWT walking distances (*p* < 0.01). See Table 2. However, after adjusting for baseline (pre-intervention) scores as covariates, ANCOVA results revealed that the SRE group achieved significantly greater improvement in the self-selected walking speed (adjusted group difference: +6.78 m/min, 95% CI [5.74–7.83]; *F* = 174.55, *p* < 0.001) and walking distance (adjusted group difference: +34.42 m, 95% CI [28.84–39.99]; *F* = 158.21, *p* < 0.001) than those of the RR group at post-intervention compared with pre-intervention. See Table 3.

### 3.3. Secondary Outcome Measures

After 8 weeks of intervention, significant improvements were observed in both groups for secondary outcome measures, including GMFM-D, E, PBS, MAS, and PCI, with all showing enhancement compared to the pre-treatment period (*p* < 0.01). See Table 2. Furthermore, after adjusting for baseline (pre-intervention) scores as a covariate, analysis of variance (ANOVA) results showed that the SRE group improved significantly more than the RR group on all of the secondary outcome measures (GMFM-D, E, PBS, MAS, and PCI) at post-intervention compared to pre-intervention (*p* < 0.001). See Table 3.

### 3.4. Correlation Analysis

**Figure 3:**
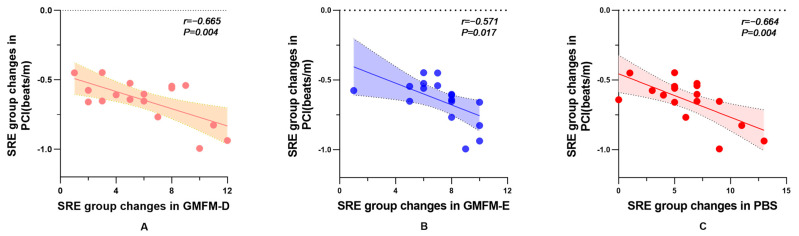
(**A**–**C**) indicate the correlation between SRE-induced changes in energy expenditure and the clinical assessment scales GMFM-D, E, and PBS, respectively. Abbreviations: SRE, soft robotic exoskeleton; GMFM-D, gross motor function measure-D zone; GMFM-E, gross motor function measure-E zone; PBS, pediatric balance scale; PCI, physiological cost index.

To further investigate the underlying mechanisms of SRE-induced changes in energy expenditure, additional correlation analyses were performed using linear regression to compare the relationship between SRE-induced changes in energy expenditure (PCI) and changes in the major clinical assessment scales (GMFM-D, E, PBS). The results showed a significant correlation between changes in energy expenditure and changes in GMFM-D (*r* = −0.665, *p* = 0.004), GMFM-E (*r* = −0.571, *p* = 0.017), and PBS (*r* = −0.664, *p* = 0.004), which suggests that reductions in energy expenditure are positively correlated with increases in GMFM-D, E, and PBS scores, respectively. See Figure 3.

### 3.5. Minimal Clinically Important Difference

Finally, the clinical significance of the intervention was investigated by comparing some of the outcome measures (10MWT, 6MWT, GMFM-D, and E) with the predefined MCID. The average improvements in 10MWT self-selected walking speed and 6MWT walking distance in the SRE group at post-intervention compared with pre-intervention were 0.17 ± 0.02 m/s and 56.82 ± 12.06 m, respectively, which were greater than the established MCIDs of the 10MWT (0.14 m/s) and 6MWT (46 m), respectively. The mean improvement of GMFM-D and E zones in the SRE group was 6.00 ± 3.32 and 7.18 ± 2.27, which was greater than the established MCIDs of the GMFM-D zones (1.8) and E zones (2.6), respectively. In contrast, the changes in 10MWT and 6MWT in the RR group were 0.05 ± 2.27 m/s and 21.78 ± 3.54 m, respectively, neither of which met the previously established MCIDs, whereas the improvements in GMFM-D and E zone scores were 3.72 ± 1.41 and 4.00 ± 1.24, respectively, which were greater than the established MCIDs but less than the improvements in the SRE group.

## 4. Discussion

The main objective of this study was to assess the effect of SRE training on lower limb motor function in children with SCP. Our main findings were as follows: First, after 8 weeks of training, both groups showed significant within-group improvements across all outcome measures. Second, the improvements observed in the SRE group were superior to those in the RR group. This indicates that SRE-assisted training has a positive impact on the lower limb motor function of SCP patients. In addition, the results of correlation analysis showed that the cause of SRE-induced reduction in energy expenditure in children may be related to the improvement of gross motor function and balance function. Overall, SRE may represent a potentially novel therapeutic device to improve motor function in children with SCP.

Achieving independent walking is a pivotal objective in both clinical and community-based rehabilitation for children with CP [34]. Walking speed and endurance are crucial factors that significantly affect the quality of life and community engagement for these children. The study’s findings revealed that post-treatment improvements were observed in both groups for the 10MWT walking speed and 6MWT walking distance (*p* < 0.01), with the SRE group demonstrating superior advancements. Specifically, the SRE group’s 10MWT walking speed and 6MWT walking distance improved by 0.17 ± 0.02 m/s and 56.8 ± 12.06 m, respectively—enhancements that not only exceeded those seen in the RR group (0.05 ± 2.27 m/s and 21.78 ± 3.54 m, respectively) but also surpassed the established MCID. These results are consistent with the findings of another randomized controlled trial, where six CP patients of levels I–III experienced increases in unassisted walking speed by 0.24 m/s and stride length by 0.17 m following four 25 min sessions of SRE-assisted gait training. However, the small sample size and short intervention duration of that study limited the strength of the evidence. Our study mitigated these limitations by employing a randomized controlled design and prolonging the intervention duration, thus yielding more robust and reliable findings. The observed benefits of SRE may be linked to principles of neural plasticity and motor learning [35,36]. The device facilitates high-intensity, repetitive gait training, enabling children to internalize correct motor patterns [14,37]. This process likely supports cortical reorganization and enhances motor function in the brain, showcasing SRE’s potential as an effective intervention for enhancing mobility in children with CP.

The primary symptoms of CP are motor and postural impairments [38], making enhancements in gross motor function and balance critical indicators of rehabilitation success. Following 8 weeks of therapy, significant improvements in GMFM-D and E zones and PBS were observed in all children participating in the study (*p* < 0.01). Notably, the GMFM-D and E zones in the SRE group improved by approximately 233% and 169%, respectively, compared with the previously set MCIDs, which was higher than the respective improvements of 107% and 54% in the RR group, aligning with findings from a previous randomized controlled trial that included 30 children with bilateral SCP. In that study, the intervention group received 20 additional training sessions with the Lokomat, a robotic gait trainer, on top of standard physical therapy and demonstrated marked improvements in gross motor function and balance over the control group, which only underwent conventional therapy [39]. However, the Lokomat is a relatively expensive and immobile device, which may limit its accessibility and practicality in some settings. These outcomes suggested that SRE, similar to the Lokomat, positively impacts gross motor function and balance in children with CP. Traditional rehabilitation robots like the Lokomat often employ impedance control strategies to apply corrective torque, ensuring the legs follow a set physiological gait path [40]. However, the predominantly passive training mode may not be fully effective for children with CP, who may struggle with active participation due to their young age or cognitive challenges. In contrast, SRE supports the child’s active walking efforts by providing assistive force, potentially enhancing engagement, and facilitating cortical reorganization and motor improvement [41]. This suggested that active training with SRE could more effectively promote lower limb motor function improvements in children with CP, offering theoretical backing for the observed benefits of SRE-assisted training in enhancing gross motor skills and balance.

The ankle joint is a significant weight-bearing part of the human body, and its stability influences the biomechanical alignment of the knee and hip, which is crucial for improving gait [42]. The MAS scores of both groups in this study showed significant improvement after treatment compared to pre-treatment (*p* < 0.01), and between-group comparisons revealed greater improvement in the SRE group (*p* < 0.001). This is consistent with the results of a previous study in which 12 children with spastic cerebral palsy underwent ankle robotic training for a period of 6 weeks, resulting in significant improvement in the spasticity of the ankle muscles [43]. However, another study presented different results. A randomized controlled trial of 41 children with cerebral palsy showed that after six weeks of passive stretching and active movement interventions on damaged ankles, there was no significant improvement in the MAS of the children in either the home or laboratory groups [44]. It is generally accepted that spasticity in the triceps surae and weakness in the ankle dorsiflexor muscles are the causes of restricted dorsiflexion in children with SCP [45], leading to a reduced plantar flexion angle during push-off and decreased propulsion, resulting in gait abnormalities. The SRE provides assistive force during plantar and dorsiflexion, similar to the effects of muscle stretching, thereby alleviating muscle spasticity through repetitive stretching of the ankle muscle groups. Therefore, the variance in experimental results may be attributed to the robotic equipment used, as well as the intensity and methodology of training. Further high-quality studies are needed to substantiate these findings in the future.

Current research findings indicated that SRE-assisted walking training improves walking distance in children. A previous study has suggested a positive correlation between increased walking distance and a reduction in the energy expenditure required to complete the task [46]. In this study, both groups of children showed significant improvements in unassisted walking energy expenditure (PCI) after 8 weeks of treatment (*p* < 0.01). Notably, the average PCI value in the SRE group decreased by 0.65 ± 0.15 beats/m, which was significantly greater than the change in the RR group of 0.34 ± 0.13 beats/m. This demonstrated that SRE-assisted gait training further reduces energy expenditure in children with SCP, aligning with the outcomes of previous studies. Although multiple previous studies [47,48] reported significant improvements in energy expenditure in CP patients wearing SRE, there has been limited research on whether these improvements persist after the SRE is removed. This study, through a randomized controlled trial, has verified that the beneficial effects of SRE-assisted gait training on energy expenditure can extend to unassisted walking, a finding similarly observed in adult stroke patients [21]. This improvement may be because muscle spasticity in children with SCP often presents in a “bloc” movement pattern. SRE-assisted walking training may help to enhance the coordination of lower limb movements, improve muscle spasticity, and normalize standing and walking postures [49], thereby reducing energy expenditure.

To further explore the mechanisms by which SRE training improves walking energy expenditure in children with SCP, we conducted correlation analyses using linear regression. These analyses examined the relationship between changes in energy expenditure (PCI) and changes in scores on key clinical assessment scales: GMFM-D, E, and PBS. The results showed a significant positive correlation between the reduction in PCI and the increase in GMFM-D, E, and PBS scores. This suggested that the decrease in energy expenditure in children is positively correlated with improvements in gross motor function and balance abilities. This trend expands upon previous research, which established a linear correlation between the reduction in energy expenditure during walking and an increase in the total GMFM score [50]. Given these correlations, our results suggested that SRE training may reduce energy expenditure in children with SCP by improving their gross motor and balance functions. Nonetheless, further high-quality studies are needed to firmly establish the causal link between SRE training and its effects on energy expenditure, gross motor function, and balance in children with SCP.

In summary, our results suggested that SRE can improve motor function in children with grade I–III SCP. As a lightweight, easy-to-use robotic exoskeleton, the SRE can be operated by non-professional healthcare workers following a brief training period. Furthermore, its cost is lower than that of traditional lower limb rehabilitation robots such as Lokomat, which facilitates the potential application of SRE in community and home settings. The next step should involve enhancing research in this area to extend its benefits to more patients. Additionally, the patient-initiated training mode of the SRE effectively reduces human-robot interaction issues and helps to minimize skin- and joint-related injuries, thereby improving the safety of rehabilitation. However, the limitations of the SRE include its low structural rigidity, reduced power, and the prerequisite that users possess certain standing and walking abilities, restricting its application among moderately and severely disabled populations.

## 5. Limitations

This study has several limitations. Firstly, the absence of follow-up observations limits our ability to determine the long-term effects of SRE-assisted training. Future studies with longer follow-up periods are needed to assess the persistence of the therapeutic benefits observed in this study. Secondly, the sample size was relatively small and precluded stratified analysis to explore potential differences in treatment response based on the severity of SCP. This limits the generalizability of our findings to children with different levels of motor impairment. Thirdly, the study did not include comprehensive gait analysis, which would have provided a more detailed understanding of how SRE training affects gait dynamics in children with SCP. Future studies should include more comprehensive gait analysis to capture additional parameters such as symmetry and joint angles. Overall, while this study provides promising evidence for the benefits of SRE-assisted walking training in children with SCP, further research is needed to address these limitations and optimize the use of SRE in clinical practice.

## 6. Conclusions

This study suggests that the combined use of SRE with conventional rehabilitation therapy may more effectively improve lower limb motor function in children with grade I–III SCP. In addition, the reduction in energy expenditure induced by the SRE may be associated with improvements in gross motor skills and balance. Overall, this study provides preliminary evidence to support the inclusion of SRE in a routine rehabilitation program for rehabilitating lower limb motor function in children with SCP.

## Figures and Tables

**Table 1:** General information of the participants.

	RR Group (n = 18)	SRE Group (n = 17)	*p*-Value
Male, n (%)	9 (50)	11 (64.7)	0.38
Age, (year) *	5.4 (2.0)	5.8 (1.8)	0.45
Height (cm) *	113.4 (13.9)	116.7 (12.7)	0.41
Weight (kg) *	22.5 (6.1)	23.1 (5.0)	0.33
Use of a walking aid, n (%)	4 (22)	3 (18)	0.74
SCP			
unilateral	10	9	0.88
bilateral	8	8	
GMFCS			
Level I	5	6	0.54
Level II	9	7	
Level III	4	4	

Abbreviations: SCP, spastic cerebral palsy; GMFCS, Gross Motor Function Classification System. * Mean (standard deviation).

**Table 2:** Pre- and post-intervention measurements and within-group differences.

Variables	RR Group (n = 18)	SRE Group (n = 17)
10MWT (m/min)		
Pre	24.7 (5.8)	24.9 (5.9)
Post	27.9 (6.5) **	34.9 (5.0) **
% Post-pre > MCID (0.14 m/s)	-	20%
6MWT (m)		
Pre	138.6 (35.2)	144.5 (35.1)
Post	160.3 (35.1) **	201.3 (44.1) **
% Post-pre > MCID (46 m)	-	23%
GMFM-D		
Pre	20.6 (10.4)	25.7 (9.4)
Post	24.3 (10.3) **	31.7 (6.9) **
% Post-pre > MCID (1.8)	107%	233%
GMFM-E		
Pre	28.5 (16.7)	33.9 (19.2)
Post	32.5 (16.8) **	41.1 (18.2) **
% Post-pre > MCID (2.6)	54%	169%
PBS		
Pre	35.0 (23.3)	34.0 (21.5)
Post	37.0 (24.3) *	39.0 (16.5) **
MAS		
Pre	2.1 (1.1)	2.1 (1.1)
Post	1.4 (1.1) **	1.3 (0.8) **
PCI (beats/m)		
Pre	1.0 (0.2)	1.0 (0.2)
Post	0.6 (0.1) **	0.3 (0.1) **

All Pre and post-values are presented as mean (SD) except for PBS values, which are presented as medians (IQR). Abbreviations: RR, conventional training; SRE, Soft robotic exoskeleton; MCID, minimal clinically important difference; 10MWT, 10 m walk test; 6MWT, 6 min walk test; GMFM-D, gross motor function measure-D zone; GMFM-E, gross motor function measure-E zone; PBS, pediatric balance scale; MAS, modified Ashworth scale; PCI, physiological cost index. * *p* < 0.01, ** *p* < 0.001, significant within-group difference.

**Table 3:** Using baseline values as covariates, adjusted for differences in outcome improvement (post-pre) in the SRE group compared to the RR group.

Outcome Measures	SRE Group vs. RR Group
10MWT (m/min)	+6.78 (5.74–7.83) ** [0.845]
6MWT (m)	+34.42 (28.84–39.99) ** [0.832]
GMFM-D	+2.98 (1.45–4.51) ** [0.330]
GMFM-E	+3.35 (2.14–4.57) ** [0.497]
PBS	+4.80 (3.17–6.43) ** [0.530]
MAS	−1.26 (0.84–1.68) ** [0.537]
PCI (beats/m)	−0.31 (−0.32–−0.25) ** [0.733]

All analyses of covariance between-group differences were presented as mean difference (d) and 95% confidence interval (95% CI), [effect size: η2]; ** *p* < 0.001, significant between-group differences. Abbreviations: 10MWT, 10 m walk test; 6MWT, 6 min walk test; GMFM-D, gross motor function measure-D zone; GMFM-E, gross motor function measure-E zone; PBS, pediatric balance scale; MAS, modified Ashworth scale; PCI, physiological cost index.

## Data Availability

The data that support the findings of this study are available on request from the corresponding author. The data are not publicly available due to privacy or ethical restrictions.
